# Maternal pre-eclampsia serum increases neurite growth and mitochondrial function through a potential IL-6-dependent mechanism in differentiated SH-SY5Y cells

**DOI:** 10.3389/fphys.2022.1043481

**Published:** 2023-01-12

**Authors:** Aaron Barron, Samprikta Manna, Colm J. McElwain, Andrea Musumeci, Fergus P. McCarthy, Gerard W. O’Keeffe, Cathal M. McCarthy

**Affiliations:** ^1^ Department of Anatomy and Neuroscience, University College, Cork, Ireland; ^2^ Department of Pharmacology and Therapeutics, University College Cork, Cork, Ireland; ^3^ Department of Obstetrics and Gynaecology, Cork University Maternity Hospital, Cork, Ireland; ^4^ Cork Neuroscience Centre, University College Cork, Cork, Ireland

**Keywords:** pre-eclampsia, neurodevelopmental disorder, autism spectrum disorder, interleukin-6, inflammation, neurite growth, mitochondria

## Abstract

**Introduction:** Pre-eclampsia (PE) is a common and serious hypertensive disorder of pregnancy, which affects 3%–5% of first-time pregnancies and is a leading cause of maternal and neonatal morbidity and mortality. Prenatal exposure to PE is associated with an increased risk of neurodevelopmental disorders in affected offspring, although the cellular and molecular basis of this increased risk is largely unknown.

**Methods:** Here, we examined the effects of exposure to maternal serum from women with PE or a healthy uncomplicated pregnancy on the survival, neurite growth and mitochondrial function of neuronally differentiated human SH-SY5Y neuroblastoma cells, which are commonly used to study neurite growth. Neurite growth and mitochondrial function are two strongly linked neurodevelopmental parameters in which alterations have been implicated in neurodevelopmental disorders. Following this, we investigated the pleiotropic cytokine interleukin-6 (IL-6) levels as a potential mechanism.

**Results:** Cells exposed to 3% (v/v) PE serum for 72 h exhibited increased neurite growth (*p* < 0.05), which was validated in the human neural progenitor cell line, ReNcell^®^ VM (*p* < 0.01), and mitochondrial respiration (elevated oxygen consumption rate (*p* < 0.05), basal mitochondrial respiration, proton leak, ATP synthesis, and non-mitochondrial respiration) compared to control serum-treated cells. ELISA analysis showed elevations in maternal IL-6 in PE sera (*p* < 0.05) and placental explants (*p* < 0.05). In support of this, SH-SY5Y cells exposed to 3% (v/v) PE serum for 24 h had increased phospho-STAT3 levels, which is a key intracellular mediator of IL-6 signalling (*p* < 0.05). Furthermore, treatment with anti-IL-6 neutralizing antibody blocked the effects of PE serum on neurite growth (*p* < 0.05), and exposure to IL-6 promoted neurite growth in SH-SY5Y cells (*p* < 0.01).

**Discussion:** Collectively these data show elevated serum levels of maternal IL-6 in PE, which increases neurite growth and mitochondrial function in SH-SY5Y cells. This rationalizes the further study of IL-6 as a potential mediator between PE exposure and neurodevelopmental outcome in the offspring.

## 1 Introduction

Pre-eclampsia (PE) is a hypertensive disorder of pregnancy affecting approximately 5% of primiparous pregnant women. PE involves new-onset hypertension on or after 20 weeks’ gestation and one of proteinuria, organ dysfunction or uteroplacental dysfunction (Brown et al., 2018; Barron et al., 2021). Well-recognized as a leading cause of maternal and neonatal morbidity and mortality, PE also has adverse consequences for the long-term health and neurodevelopmental trajectories of exposed offspring (Wu et al., 2009; Andraweera and Lassi, 2019; Li et al., 2021). This includes an increased risk of neurodevelopmental disorders, particularly autism spectrum disorder (ASD), attention-deficit/hyperactivity disorder (ADHD), and intellectual disability (ID) (Maher et al., 2018; Sun et al., 2020; Greca et al., 2021; Wang et al., 2021). In addition, recent neuroimaging studies have revealed alterations in brain structure, function and metabolites of children prenatally exposed to PE (Rätsep et al., 2016; Figueiró-Filho et al., 2017; Mak et al., 2018; Katsuki et al., 2021; Xing et al., 2021).

For these reasons, there has been significant interest in using rodent models to examine the brain and behavior of offspring prenatally exposed to a PE-like state *in utero*. These have yielded valuable insights into the effects of exposure to PE-like environment on mammalian neurodevelopment, which include alterations in neurogenesis and gliogenesis, regional brain volumes, forebrain transcriptional profile, and pronounced behavioral deficits (Liu et al., 2016; Ijomone et al., 2020; Gumusoglu et al., 2021; Rains et al., 2021). However, there is a need to understand whether exposure to PE affects neuronal development at a single cell level, particularly in human cells, in order to understand the potential mechanisms involved. For example, some studies have reported that exposure to PE serum increases neurite growth and branching in embryonic day (E)18 rat primary cortical neurons (Curran et al., 2018); yet others have shown that secreted factors from the PE placenta *reduce* neurite growth in E18 cortical neurons, alter neurotransmitter receptor expression and enhance astrogliogenesis (Scott et al., 2018). Thus, there is a need for further studies that explore the physiological effects, and molecular mechanisms, of PE exposure on developing neurons.

This study aimed to assess the effects of PE exposure on neurite growth and mitochondrial function, two important neurodevelopmental parameters known to be implicated in neurodevelopmental disorders, particularly ASD (Gu et al., 2013; Hashimoto et al., 2016; Barron et al., 2021). These are two tightly linked processes: neurites are rich in mitochondria; mitochondrial-derived reactive oxygen species derived are key regulators of neurodevelopmental processes, including neurite growth; and a significant proportion of cellular ATP, generated by mitochondria in the neurite and growth cone, is used for actin polymerization, the chief mechanism responsible for neurite growth (Smith and Gallo, 2018; Wilson et al., 2018). Therefore, the experiments described here examined whether serum from women with PE or women with healthy uncomplicated pregnancies (controls) differentially affect neurite growth and mitochondrial function in neuronally-differentiated–SH-SY5Y cells, a human neuroblastoma cell line commonly used to study neurite growth *in vitro* (Kovalevich and Langford, 2013). The use of human sera was chosen to identify whether there are maternal circulating factors in PE that affect neuronal development.

While the physiological mechanisms underlying the association between PE and offspring neurodevelopment are yet to be discerned, one candidate mechanism may be the sustained maternal immune activation (MIA) which is a prominent feature of PE (Sharma et al., 2007; Cornelius, 2018; Aggarwal et al., 2019; Barron et al., 2021). MIA is known to adversely affect neurodevelopment both directly *via* the effects of cytokines on neurodevelopmental processes in the fetal brain (Jarskog et al., 1997; Nolan et al., 2011; Crampton et al., 2012), and indirectly *via* non-canonical mechanisms through which MIA-induced alterations of maternal physiology create a sub-optimal *in utero* environment for the fetus (Shi et al., 2005; Zuckerman and Weiner, 2005; Brown et al., 2014; Straley et al., 2017; Barron et al., 2021).

Specifically, the cytokine interleukin-6 (IL-6) may play a significant role in this association. Elevated maternal IL-6 is associated with altered structural and functional brain connectivity in the offspring (Spann et al., 2018; Rasmussen et al., 2019), and the adverse effects of MIA on offspring brain and behavior in animal models are dependent on maternal or placental IL-6 (Smith et al., 2007; Gumusoglu et al., 2017; Wu et al., 2017). The phenotypic effects of IL-6 signaling, acting through phospho-activation of the transcription factor signal transducer and activator of transcription 3 (STAT3) at Tyr_705_, are pleiotropic, although in neurons it typically exerts a neurogenic, neuritogenic and neurotrophic effect–several studies have identified a role for IL-6-STAT3 signaling in promoting neuronal differentiation and survival, and enhancing neurite outgrowth, axon regeneration and synaptogenesis, in various neuronal models (März et al., 1997; Bissonnette et al., 2004; Miao et al., 2006; Zhou and Too, 2011; Yang et al., 2012; Leibinger et al., 2013; Su et al., 2020; Kummer et al., 2021; Mirabella et al., 2021). Importantly, STAT3 is also known to stimulate mitochondrial activity (Gough et al., 2009; Zhou and Too, 2011; Yang et al., 2015; Luo et al., 2016; Su et al., 2020).

Maternal IL-6 is reportedly elevated in PE (Aggarwal et al., 2019; Gencheva et al., 2021), it crosses both the placental and blood-brain barriers (Zaretsky et al., 2004; Banks, 2005) and is increased in the umbilical cord blood of neonates exposed to PE (Tosun et al., 2010). For these reasons, we measured IL-6 in serum and placental explant supernatants in PE and hypothesized that elevated IL-6 in PE would increase neurite growth and mitochondrial respiration in neuronally differentiated SH-SY5Y cells.

## 2 Materials and methods

### 2.1 Patient enrolment and serum collection

Pre-eclampsia patients and controls were recruited from Cork University Maternity Hospital, Cork, Ireland, as part of the COMRADES Study, a non-interventional cohort study of nulliparous singleton pregnancies with the aim of characterizing the immune cell profile of women with PE. PE cases (*n* = 18) were defined as sustained hypertension (with systolic blood pressure (BP) ≥ 140 or diastolic BP ≥ 90 on at least 2 occasions at least 4 h apart) with significant quantified proteinuria (>300 mg protein on 24 h collection, urine protein creatinine >30 mg/mmol or +3 Dipstick Proteinuria) as per International Society for the Study of Hypertension in Pregnancy guidelines (Brown et al., 2018). Matched selected controls (*n* = 18) were taken from healthy pregnant women who had uncomplicated pregnancies which were defined as pregnancies not affected by PE, preterm birth or fetal growth restriction and delivered at >37 weeks. All control blood pressure readings were <140 and/or <90 mmHg prior to the delivery. Controls were matched with the PE cases for maternal age, body mass index (BMI) and gestational age. All women were delivered by prolabor elective Caesarean section for reasons such as breech presentation. Fasting blood samples were taken the morning of the scheduled elective Caesarean section. Serum samples were collected in BD EDTA Vacutainer tubes, placed on ice, and centrifuged once at 2,400 g for 10 min, followed by once at 2,000 g for 10 min, at 4°C according to a standardized protocol. Serum samples were stored at −80°C until analysis. The COMRADES study was conducted according to the guidelines laid down in the Declaration of Helsinki, and all the procedures were approved by the Clinical Research Ethics Committee of the Cork Teaching Hospitals [ECM4 (ff) 04/12/18], and all women provided written informed consent. Clinical data from women with pre-eclampsia and matched healthy controls are shown in Table 1.

**TABLE 1:** Maternal clinical characteristics for all control and patient mothers enrolled in the current study. Mean ± SD. Mean Arterial blood pressure was calculated as MAP = (2 × diastolic) + systolic/3.

	Uncomplicated controls (*n* = 18)	Pre-eclampsia (*n* = 18)	*p*-value
Maternal age (years)	35.2 ± 4.5	36.5 ± 6.6	0.6909
Maternal BMI (kg/m^2^)	25.2 ± 5.2	30.8 ± 7.9	0.1731
Mean arterial blood pressure in 1st trimester (mm Hg)	78.8 ± 6.5	88 ± 6.2	0.0310*
Gestational age at delivery (weeks)	38.7 ± 1	36.5 ± 1.5	0.0160*
Fetal birthweight (g)	3,339.7 ± 368.2	2,836.7 ± 882.4	0.2266

### 2.2 Cell culture, differentiation and treatments

Human neuroblastoma SH-SY5Y cells (ATCC) were cultured in Dulbecco’s modified Eagle’s (DMEM)/Nutrient Mixture F-12 Ham’s medium, supplemented with 2 mM·L-glutamine, 1% penicillin-streptomycin, and 10% fetal bovine serum (FBS) (all from Sigma Aldrich) and maintained in a T75 culture flask (Sarstedt) at 37°C and 5% CO_2_. Media was changed every 3 days and cells were passaged and/or plated for experiments once they were ∼80% confluent. In all experiments except where otherwise indicated, 10 μM retinoic acid (RA, Sigma Aldrich) was added daily for the experimental duration to induce partial neuronal differentiation, concomitant with other experimental interventions.

For some experiments, full neuronal differentiation was achieved by adapting a 12-day protocol described by Taylor-Whiteley et al., 2019 (Taylor-Whiteley et al., 2019). Briefly, SH-SY5Y cells were cultured in Dulbecco’s modified Eagle’s (DMEM) high glucose medium, which included 2 mM L-glutamine, and supplemented with 1% penicillin-streptomycin, 1 mM sodium pyruvate and 10% FBS (all from Sigma Aldrich). Cells were seeded in a 24-well plate at 10,000 cells per well in complete high glucose media +10% FBS and treated daily with 10 μM RA for 5 days. After 5 days, cells were washed once in serum-free, high-glucose media and then the media was changed to serum-free, high-glucose media. Cells were then treated daily with 50 ng/mL brain-derived neurotrophic factor (BDNF, Peprotech) for a further 7 days before analysis.

The human neural progenitor cell line ReNcell® VM (Sigma) was used to validate findings. Cells were cultured in ReNcell® Maintenance Medium supplemented 20 ng/mL EGF (epidermal growth factor) and FGF2 (fibroblast growth factor 2) (all from Sigma Aldrich) and maintained in a T75 culture flask (Sarstedt) at 37°C and 5% CO_2_. Cells were seeded at 7,500 cells per well in a laminin-coated 96-well plate. 24 h after seeding, cells were washed, and media changed to ReNcell® Maintenance Medium without EGF and FGF2—the restriction of growth factors initiates spontaneous differentiation to neurons. Cells were differentiated for 7 days.

For all experiments, except where otherwise indicated, treatments were 2 h after plating, and analyses were performed 72 h after first treatment. Final concentrations used were: 10 μM RA or 50 ng/mL BDNF added daily during differentiation; 3% (v/v) maternal serum from women with PE or normotensive pregnant women, added once (Curran et al., 2018); 20 ng/mL recombinant IL-6 (Peprotech), added daily (Qian et al., 2014; Sackmann et al., 2017; Marko et al., 2020); and 0.5 μg/mL anti-IL-6 function-blocking antibody (R&D Systems, MAB 206), incubated with sera or IL-6 for 1 h at room temperature before respective treatments. Of the total *n* = 18 control and *n* = 18 PE sera, smaller samples were selected randomly or based on availability, and sample sizes for each experiment are detailed in the figure legends. All experiments involved equal numbers of control and PE sera, where one PE serum sample was equal to one independent replicate (n).

### 2.3 Quantification of IL-6 in maternal serum and placental explant supernatants

IL-6 was examined using the U-PLEX Biomarker Group 1 Human Assays K15067L-1 immunoassay (Mesoscale Diagnostics, United States). All standards and serum and placenta explant supernatant samples were run in duplicate. Plates were prepared according to manufacturer’s instructions and analyzed on the Meso QuickPlex SQ 120. Results were generated as calculated concentration means on the Mesoscale (MSD) Discovery Workbench 4.0 assay analysis software. The MSD analysis software determines individual cytokine concentrations from electro-chemiluminescent signals *via* backfitting to the calibration curve. IL-6 concentration is presented in pg/mL.

### 2.4 Neurite length measurements

For neurite growth measurements, cells were plated at a density of 12,500 cells/cm^2^ and live-cell imaging was performed after 72 h using either fluorescent microscopy following 1 h incubation with the vital cell dye Calcein-AM (Sigma Aldrich) at 0.4 μg/mL, or phase contrast, at × 20 magnification using an Olympus I ×71 inverted microscope. Five non-overlapping fields were acquired per well with a DP72 camera, and neurites were traced to calculate neurite length using ImageJ. In all cases the analyses were performed in a blinded fashion.

### 2.5 Scratch wound assay

A scratch wound assay experiment was used to assess cell migration. SH-SY5Y cells were grown until confluent for 72 h. A single, straight scratch was made through the cell monolayer using a P200 pipette tip and the media was then changed. The wound was imaged using phase contrast microscopy at ×10 magnification on an Olympus I ×71 inverted microscope at three distinct locations in each well at the following timepoints post-scratch: 0 h, 24 h, 48 h, and 72 h. The mean wound width was measured at each time point using ImageJ, and this was used to calculate the rate of wound closure as a measure of cell migration.

### 2.6 Oxidative stress measurement

Oxidative stress was assessed using the fluorescent cell dye CellROX^TM^ Green Reagent (Invitrogen), according to manufacturer’s guidelines. Briefly, cells were incubated with 5 μM CellROX^TM^ Green Reagent at 37°C for 30 min, then washed once in PBS and imaged live in PBS at ×20 magnification using FITC fluorescent channel, on an Olympus I X71 inverted microscope. Five non-overlapping fields were acquired per well with a DP72 camera. The mean fluorescence intensity of five cells per field minus adjacent background was measured using ImageJ.

### 2.7 Cytotoxicity assay

Cytotoxic cell damage was determined using the CyQUANT^TM^ LDH Cytotoxicity Assay Kit (Invitrogen), which measures cytotoxicity based on extracellular lactate dehydrogenase (LDH) activity, according to manufacturer’s guidelines. Briefly, media was collected at the end of each experiment and centrifuged to remove any remaining cells or debris, and the supernatant was collected and used for the assay. 50 μL of the medium was combined with 50 μL of the reaction mixture in a flat-bottomed, 96-well plate and incubated for 30 min at room temperature in darkness. The reaction was terminated with 50 μL of stop solution and absorbance at 680 nm measured and subtracted from absorbance at 490 nm.

### 2.8 Mitochondrial respiration

Mitochondrial function and metabolism was assessed using the Seahorse XF96 Mito Stress Test (Agilent Technologies). Optimal seeding density for SH-SY5Y cells for 3 days was determined to be 40,000 cells per well. For all subsequent experiments, cells were seeded at 40,000 cells/well in a XF96 culture plate, with 4 corner wells left empty for background correction. One hour before the assay, media was changed to Seahorse XF DMEM media, supplemented with 2 mM L-glutamine, 1 mM pyruvate and 10 mM glucose, and cells were allowed to equilibrate at 37°C and 0% CO_2_ for 1 h. After calibration, oxygen consumption rate (OCR) was measured by the Seahorse XF96 Analyzer and recorded with XF Wave software 1.4.2. at 12 distinct timepoints over the course of an 80-min run: three times at basal respiration; three times following injection of 2.5 μM oligomycin to inhibit complex V; three times following injection of 2 μM of the ionophore carbonyl cyanide-p-trifluoromethoxyphenylhydrazone (FCCP) to uncouple the H^+^ gradient at the inner mitochondrial membrane; and three times following injection of 0.5 μM each of rotenone and antimycin A, to inhibit complexes I and III, respectively. After completion of the assay, cells were lysed in 1X RIPA buffer and total protein quantified by bicinchoninic acid (BCA) assay, and OCR values normalized to protein concentration per well. From normalized OCR values, the following respiratory parameters were calculated: basal respiration, proton leak, maximal respiration, non-mitochondrial respiration, ATP production and spare respiratory capacity.

### 2.9 Mitochondrial superoxide, biomass, and membrane potential

Mitochondrial superoxide, mitochondrial biomass and mitochondrial membrane potential were measured using the fluorescent dyes MitoSOX^TM^ Red (2.5 μM, Invitrogen), MitoGreen (200 nM, Promocell) and MitoTracker^TM^ Deep Red FM (50 nM, Invitrogen), respectively. For all three dyes, cells were seeded at 37,500 cells/cm^2^ for 72 h, and then incubated with the dye at 37°C for 30 min, as per manufacturers’ guidelines. The dye was then removed, and cells detached with trypsin-EDTA and analyzed live in fluorescence-activated cell sorting (FACS) buffer containing PBS, 2% FBS and 2 mM EDTA. Mean fluorescence intensity was determined by FACS, using the BD LSRII Flow Cytometer (BD Biosciences). 20,000 events were measured for MitoSOX^TM^ Red and MitoGreen, and 10,000 for MitoTracker^TM^ Deep Red FM to determine the geometric mean representing mean fluorescence intensity.

### 2.10 Immunocytochemistry

Cells seeded at 12,500 cells/cm^2^ for 72 h were fixed for immunostaining in 4% PFA and preserved in 0.02% PBS-Triton × (PBS-T). Non-specific binding was blocked by incubating the cells in 5% BSA at room temperature for 1 h. Cells were then incubated at 4°C overnight with a primary antibody against βIII tubulin (1:1,000 (0.5 μg/mL), R&D Systems MAB1195). After overnight incubation, cells were washed in PBS-T and incubated at room temperature for 2 h with goat anti-mouse alexa fluor 594 secondary antibody (1:500, Invitrogen A11005). Cells were washed in PBS-T, counterstained with DAPI and imaged at ×20 magnification on an Olympus IX71 inverted microscope using the appropriate fluorescent filter (DAPI or TXRED). Five non-overlapping fields were acquired per well with a DP72 camera and mean fluorescence intensity was determined using ImageJ.

### 2.11 Western blot

Confluent cells were lysed in 1X radioimmunoprecipitation assay (RIPA) buffer, centrifuged at 14,000 × g for 10 min, and supernatants were stored at −80°C prior to Western blot analysis. Protein concentration was determined using a PierceTM bicinchoninic acid (BCA) assay (ThermoFisher), and xxμg protein from each cell lysate was separated by SDS-PAGE (sodium dodecyl sulphate–polyacrylamide gel electrophoresis) and transferred onto a methanol-activated PVDF membrane (Millipore). The membrane was blocked in 5% BSA for 1 h at room temperature and incubated at 4°C overnight with primary antibody against βIII tubulin (1:1,000 (0.5 μg/mL), R&D Systems MAB1195), STAT3 (1:1,000 (0.05 μg/mL), Cell Signaling Technology mAb No. 9139), p-STAT3 (1:2000 (0.05 μg/mL), Cell Signalling Technology mAb No. 9145) or GAPDH (1:1,000 (0.2 μg/mL), Santa Cruz Biotechnology sc-47724). After overnight incubation, the membrane was washed in 0.1% TBS-Tween (TBS-T) and incubated at room temperature for 1 h with goat anti-rabbit secondary antibody (1:5,000, Cell Signaling Technology mAb No. 7074) or HRP-conjugated mouse IgG_κ_ light chain binding protein (1:2000 Santa Cruz Biotechnology Product No. sc-516102). Membrane was washed in TBS-T and developed using Pierce™ ECL Western Blotting Substrate (Thermo Scientific) and the Fujifilm LAS3000 luminescent image analyzer.

### 2.12 Statistical analysis

All statistical analyses were performed using Graphpad Prism 9. Statistical significance was set at *p* < 0.05, and the statistical tests applied to the data were Student’s unpaired two-tailed t-test, one- and two-way ANOVA or mixed effects model as appropriate, with any statistically significant main effects further analysed using Fisher’s least significant difference (LSD) post-hoc test. All data are expressed as the mean with standard error of the mean (SEM) where indicated. Where data followed a non-parametric distribution, Mann-Whitney test was used. Results from t-tests are reported as t_x_ = y, *p* = z, where x is the degrees of freedom, y is the t-statistic, and z is the *p*-value; results from F-tests are reported as F_a,b_ = c, *p* = d, where a is the between-groups degrees of freedom, b is the within-groups degrees of freedom, c is the F-statistic, and d is the *p*-value.

## 3 Results

### 3.1 Exposure to PE serum increases neurite growth in differentiated SH-SY5Y cells

**FIGURE 1:**
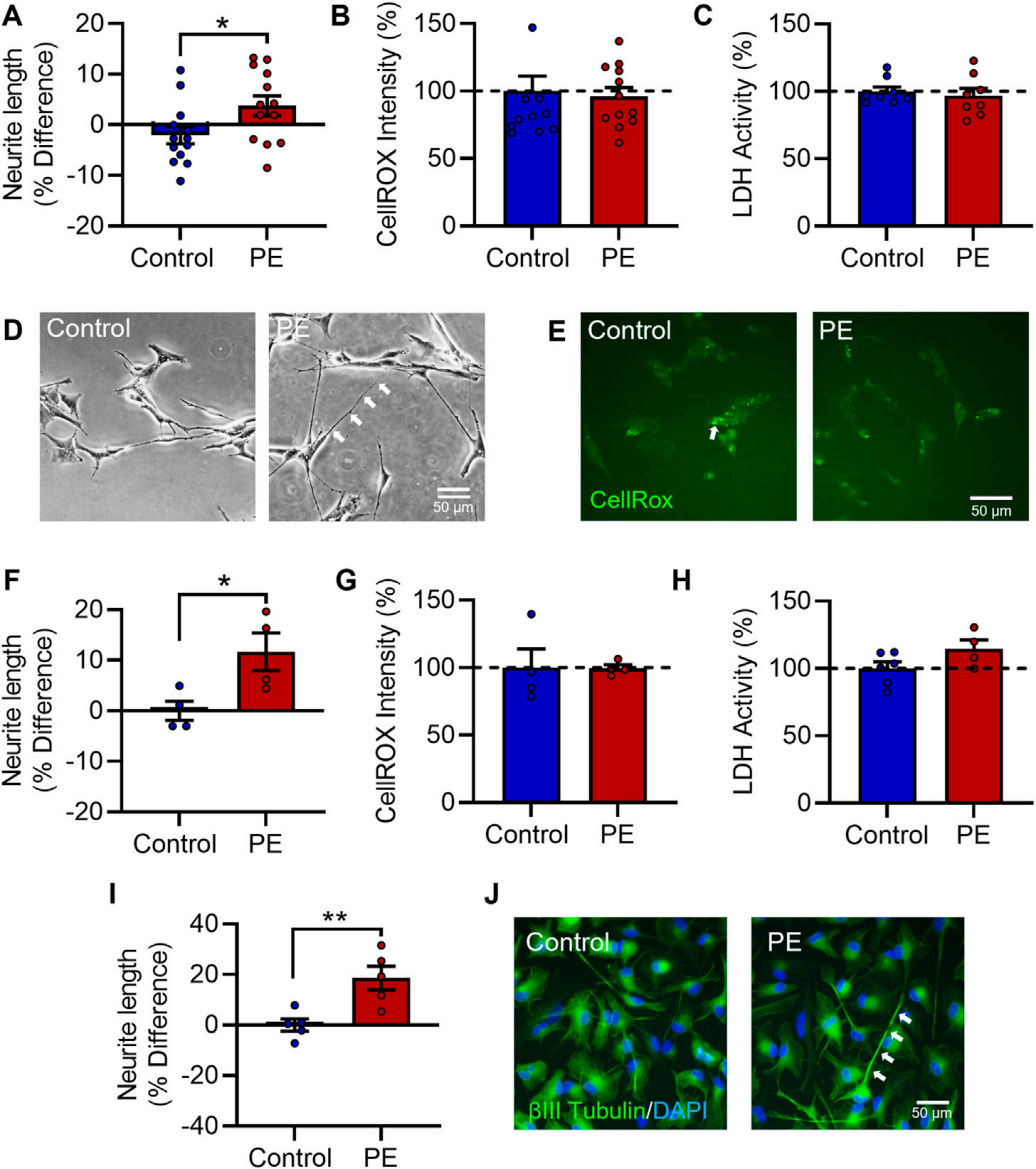
Pre-eclampsia serum increases neurite growth in differentiated SH-SY5Y Cells. RA-differentiated SH-SY5Y cells were treated with 3% (v/v) serum from pre-eclamptic patients (PE) or normotensive pregnant controls for 72 h. **(A–C)** Graphs of **(A)** neurite growth, **(B)** CellROX™ Green Reagent fluorescent intensity as a measure of oxidative stress, and **(C)** extracellular LDH activity as a measure of cytotoxicity. **(D,E)** Representative photomicrographs of **(D)** neurite growth, imaged under phase contrast, and **(E)** CellROX™ green reagent fluorescent intensity, 72 h after serum treatment. **(F,G)** SH-SY5Y cells were neuronally differentiated with 10 µM RA daily for 5 days and 50 ng/mL BDNF daily for 7 days, with 3% (v/v) serum from pre-eclamptic patients (PE) or normotensive pregnant controls for the last 3 days *in vitro*, and assessed for **(F)** neurite growth, **(G)** oxidative stress, and **(H)** cytotoxicity. **(I,J)** Graph and representative photomicrographs of neurite growth in serum-exposed ReNcell® VM cells stained for βIII tubulin (green) reactivity and bisbenzimide (blue). Data are mean +SEM from thirteen, twelve, or eight serum samples per group for **(A–C)**, respectively (*n* = 13; *n* = 12; *n* = 8); four per group for F-H (*n* = 4); and five per group for **(I,J)** (*n* = 5). Student’s unpaired *t*-test for A, **(F–I)**, Mann-Whitney test for **(B–C)** (* *p* < 0.05, ** *p* < 0.01 vs. control).

SH-SY5Y cells were differentiated with 10 μM RA for 72 h (Supplementary Figures S1A–E). To examine the effects of maternal PE serum, RA-differentiated SH-SY5Y cells were co-treated with 3% (v/v) maternal serum from women with PE or women with healthy uncomplicated pregnancies, that were matched for maternal and gestational age and maternal BMI. Neurite growth was examined at 72 h post serum treatment. Exposure to PE serum significantly increased neurite growth compared to controls (t_24_ = 2.230, *p* < 0.05) (Figures 1A, D). There was no significant change in oxidative stress (U = 65, Med_1_ = 82.24, n_1_ = 8, Med_2_ = 90.21, n_2_ = 8, *p* = 0.713) (Figures 1B, E) or cytotoxicity (U = 26, Med_1_ = 96.42, n_1_ = 8, Med_2_ = 93.78, n_2_ = 8, *p* = 0.574) (Figure 1C), as measured by CellROX^TM^ Green Reagent fluorescent intensity or extracellular LDH activity, respectively. To validate these findings, we next used a 12-day RA + BDNF differentiation protocol which promotes longer and more complex neurite growth. Similarly, RA + BDNF-differentiated cells treated with PE serum significantly increased neurite growth relative to controls (t_6_ = 2.776, *p* < 0.05) (Figure 1F), without changes in oxidative stress (t_6_ = 0.028, *p* = 0.978) (Figure 1G) or cytotoxicity (t_8_ = 1.797, *p* = 0.110) (Figure 1H).

Lastly, to confirm this phenotype in a more neuronal model, the human neural progenitor cell line ReNcell® VM was differentiated for 7 days by restriction of the growth factors EGF and FGF2, and exposed to maternal serum for the last 3 days *in vitro*. As in SH-SY5Y cells, PE serum increased neurite growth in differentiating ReNcell® VM cells (t_8_ = 3.542, *p* < 0.01) (Figures 1I, J). Collectively, these data show exposure to PE serum increases neurite growth which is not secondary to any changes in oxidative stress or viability in differentiated human neuroblastoma and human neural progenitor cells.

### 3.2 PE serum increases the oxygen consumption rate in differentiated SH-SY5Y cells

**FIGURE 2:**
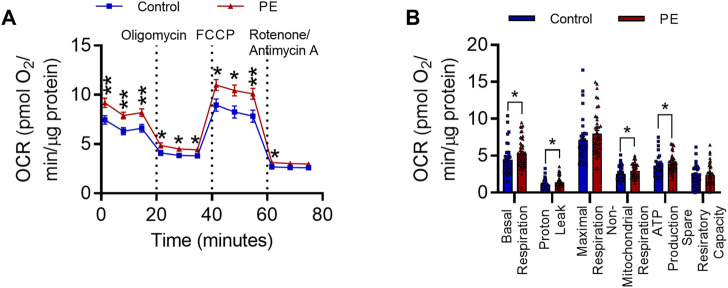
Pre-eclampsia serum alters mitochondrial function in differentiated SH-SY5Y Cells. **(A)** Oxygen consumption rate during 80-min Seahorse XF Mito Stress Test. Mean OCR values are normalized to protein content per well. **(B)** Graph representing individual parameters of respiration, calculated from the values plotted in I. Data are mean +SEM from *N* = 10 serum samples for each group with *n* = 1–5 wells per sample for I and J, or, expressed as percentage of the control. [*p < 0.05, **p < 0.01 vs. control. Mixed effects model and *post-hoc* Fisher’s least significant difference (LSD) test for **(A)**, mann-whitney test for **(B)**].

As PE serum has previously been shown to induce alterations in mitochondrial function in endothelial cells (McCarthy and Kenny, 2016), we next determined whether exposure to PE serum affects mitochondrial function in SH-SY5Y cells. To do this we performed bioenergetic state analysis of the oxygen consumption rate (OCR) in RA-differentiated SH-SY5Y cells treated with 3% (v/v) serum from women with PE or women with healthy uncomplicated pregnancies for 72 h. Cells treated with PE serum had significantly elevated OCR relative to those treated with control serum (F_1,96_ = 10.01, *p* < 0.01) (Figure 2A). This equated to significant increases in basal respiration (U = 710, Med_1_ = 4.149, n_1_ = 42, Med_2_ = 4.985, n_2_ = 49, *p* < 0.05), proton leak (U = 723, Med_1_ = 0.9654, n_1_ = 42, Med_2_ = 1.199, n_2_ = 49, *p* < 0.05), non-mitochondrial respiration (U = 758, Med_1_ = 2.404, n_1_ = 42, Med_2_ = 2.932, n_2_ = 49, *p* < 0.05), and ATP synthesis (U = 727, Med_1_ = 3.117, n_1_ = 42, Med_2_ = 3.901, n_2_ = 49, *p* < 0.05) (Figure 2B). This effect was not accompanied by changes in mitochondrial superoxide (t_5_ = 0.3103, *p* = 0.769) (Supplementary Figure S2A), biomass (t_5_ = 1.233, *p* = 0.276) (Supplementary Figure S2B), or membrane potential (t_5_ = 1.498, *p* = 0.1945) (Supplementary Figure S2C), measured with the fluorescent mitochondrial dyes MitoSOX^TM^ Red, MitoGreen and MitoTracker^TM^ Deep Red FM, respectively. Taken together these data indicate exposure to maternal PE serum leads to elevations in mitochondrial and non-mitochondrial oxygen consumption.

### 3.3 Elevated levels of maternal IL-6 in PE

**FIGURE 3:**
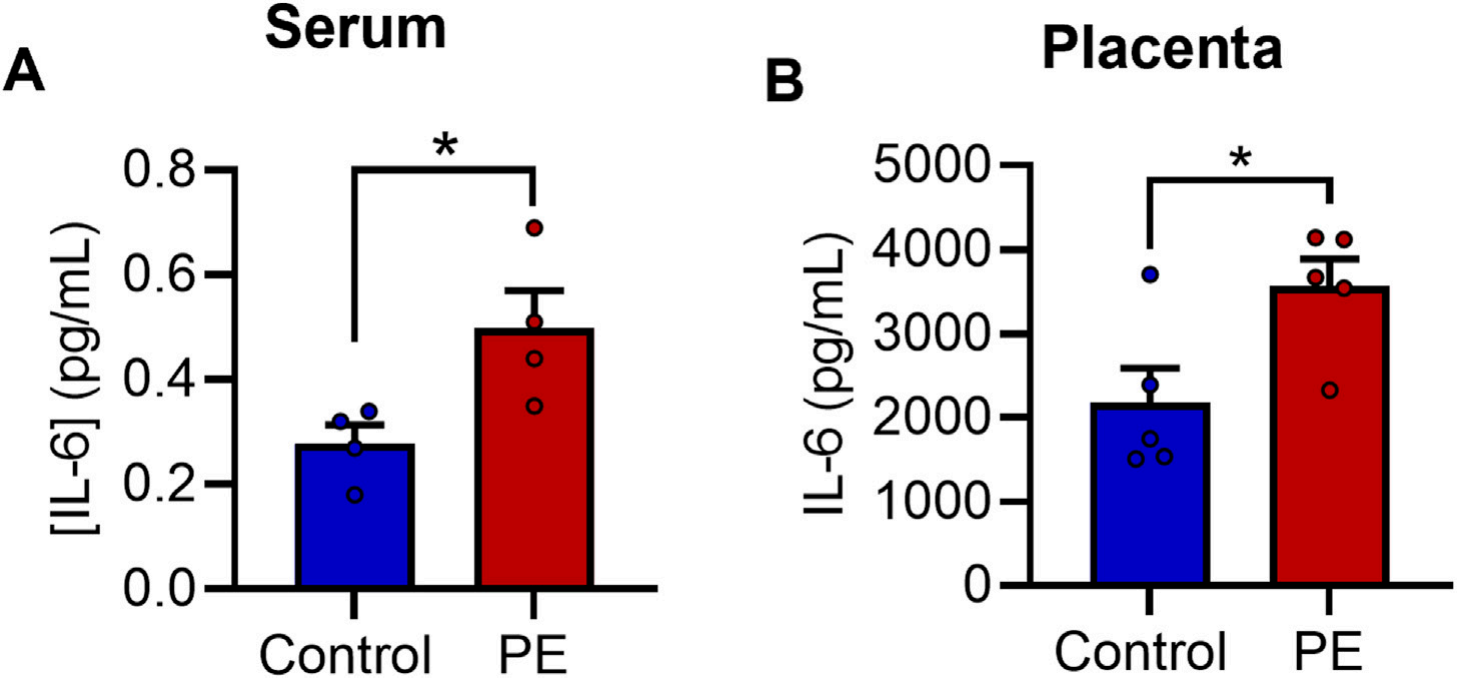
IL-6 is elevated in pre-eclampsia serum. Evaluation of [IL-6] in **(A)** maternal serum samples and **(B)** placental explant secretions. Data are mean +SEM from four samples per group for A and five for B (*n* = 4–5). (*p < 0.05 vs. control. Student’s unpaired *t*-test).

We next sought to gain insight into the molecular basis of increased neurite growth and altered mitochondrial function following exposure to PE serum. Due to previous reports of elevated IL-6 in PE (Aggarwal et al., 2019; Gencheva et al., 2021) and the known effects of IL-6 on neuronal development (März et al., 1997; Kummer et al., 2021; Mirabella et al., 2021), we postulated that IL-6 may be involved in mediating these effects. We therefore examined the levels of IL-6 in maternal sera and placental explant secretions using an immunoassay in a cohort of women with PE and uncomplicated controls. The levels of IL-6 were significantly elevated by 79% in women with PE compared to serum from healthy pregnant women (0.4975 ± 0.0357 pg/mL vs. 0.2775 ± 0.0720 pg/mL, t_6_ = 2.737, *p* < 0.05) (Figure 3A). Similarly, levels of IL-6 were also elevated in placental explants from women with PE when compared to controls respectively (3,560.7 ± 330 pg/mL vs. 2,178.3 ± 413 pg/mL, t_8_ = 2.614, *p* < 0.05) (Figure 3B).

### 3.4 IL-6 signaling is stimulated in pre-eclampsia serum-treated RA-differentiated SH-SY5Y cells and is required for increased neurite growth

**FIGURE 4:**
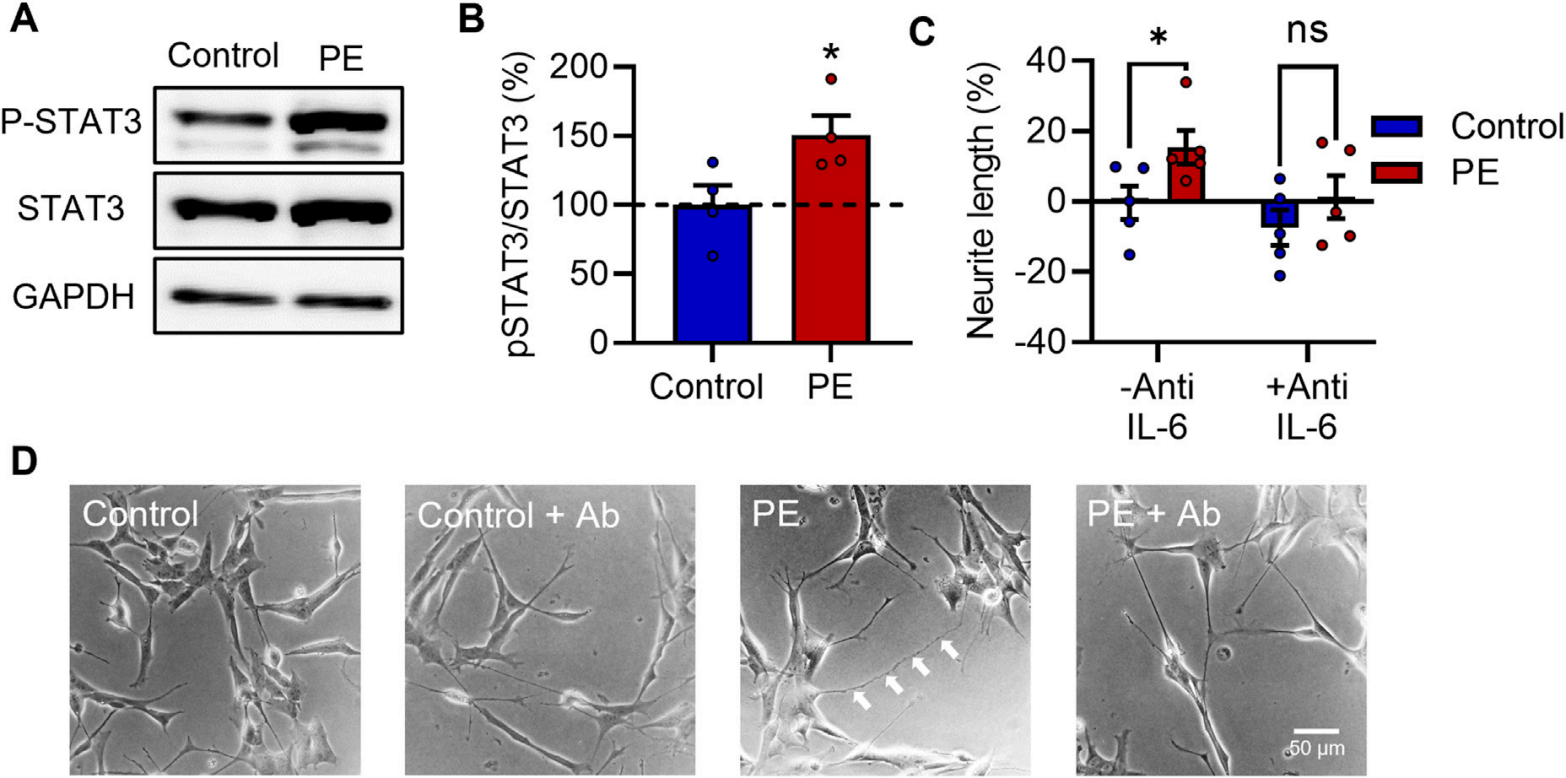
IL-6 signaling is stimulated in pre-eclampsia serum-treated RA-differentiated SH-SY5Y cells and is required for increased neurite growth. **(A, B)** Protein expression of p-Tyr_705_ STAT3 relative to total STAT3 in RA-differentiated SH-SY5Y cells treated with 3% (v/v) serum for 24 h. **(C, D)** Graph and representative photomicrographs of neurite growth 72 h after serum treatment with or without an anti-IL-6 function-blocking antibody. Data are mean +SEM from four serum samples per group for B, or five for C (*n* = 4–5). (*p < 0.05 vs. control. Student’s unpaired *t*-test for B, 2-way ANOVA and *post-hoc* Fisher’s least significant difference (LSD) test for **(C)**.

IL-6 activates the JAK/STAT signaling pathway resulting in phosphorylation of the transcription factor signal transducer and activator of transcription 3 (STAT3) at Tyr_705_ (Carpenter and Lo, 2014). To examine whether exposure to PE serum stimulated the IL-6 signaling pathway, RA-differentiated SH-SY5Y cells were treated with 3% (v/v) maternal serum for 24 h and were then assessed for phosphorylation of STAT3 at Tyr_705_ by Western blot. Expression of p-Tyr_705_ STAT3 relative to total STAT3 was significantly increased by 50% in cells treated with PE serum vs. control serum (t_6_ = 2.499, *p* < 0.05) (Figures 4A, B).

Several studies have identified a role for IL-6-STAT3 signaling in enhancing neurite outgrowth in various neuronal models (März et al., 1997; Bissonnette et al., 2004; Miao et al., 2006; Zhou and Too, 2011; Yang et al., 2012; Leibinger et al., 2013; Su et al., 2020; Kummer et al., 2021; Mirabella et al., 2021). Next, to determine whether IL-6 signaling is necessary for the increased neurite growth caused by PE serum, RA-differentiated SH-SY5Y cells were treated with 3% (v/v) serum for 72 h in the presence of a function-blocking anti-IL-6 antibody (anti-IL-6). Anti-IL-6 attenuated the neuritogenic effects of PE serum—2-way ANOVA revealed a main effect for PE vs. control sera (F_1,16_ = 5.519, *p* < 0.05) (Figures 4C, D), while *post-hoc* analyses showed a significant difference specifically between control and PE groups in the absence of anti-IL-6 (*p* < 0.05), but in its presence (*p* = 0.252), suggesting that IL-6 is required for the increased neurite growth seen in cells exposed to PE serum.

### 3.5 IL-6 increases neurite growth in differentiated SH-SY5Y cells

**FIGURE 5:**
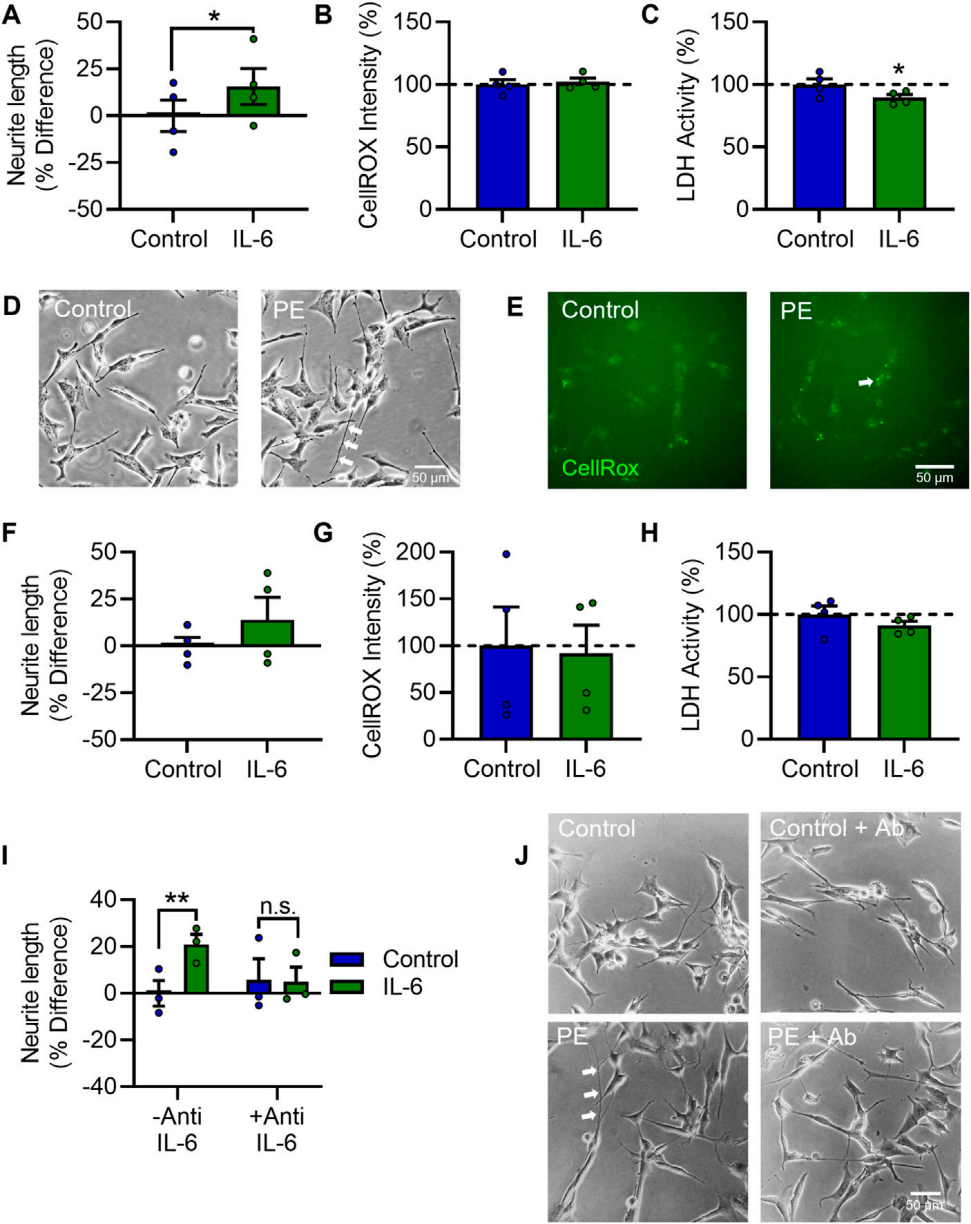
IL-6 increases neurite growth and enhances mitochondrial activity in differentiated SH-SY5Y Cells. RA-differentiated SH-SY5Y cells were treated with 20 ng/mL IL-6 daily for 72 h. **(A–C)** Graphs of **(A)** neurite growth, **(B)** CellROX™ Green Reagent fluorescent intensity as a measure of oxidative stress, and **(C)** extracellular LDH activity as a measure of cytotoxicity. **(D, E)** Representative photomicrographs of **(D)** neurite growth, imaged under phase contrast, and **(E)** CellROX™ Green Reagent fluorescent intensity, 72 h after serum treatment. **(F–H)** Graphs of **(F)** neurite growth, **(G)** oxidative stress, and **(H)** cytotoxicity in cells differentiated with 10 µM RA daily for 5 days and 50 ng/mL BDNF daily for 7 days, with or without 20 ng/mL IL-6, added daily for the last 3 days *in vitro.* **(I,J)** Graph and representative photomicrographs of neurite growth 72 h after IL-6 treatment with or without an anti-IL-6 function-blocking antibody. Data are mean +SEM from four independent experiments for **(A–C)**, **(F–H)**, or three for I (*n* = 3–4), expressed as percentage of the control. (*p < 0.05; **p < 0.01 vs. control. Student’s paired *t*-test for A–C, F–H, 2-way ANOVA and *post-hoc* fisher’s least significant difference test (LSD) for .I.

**FIGURE 6:**
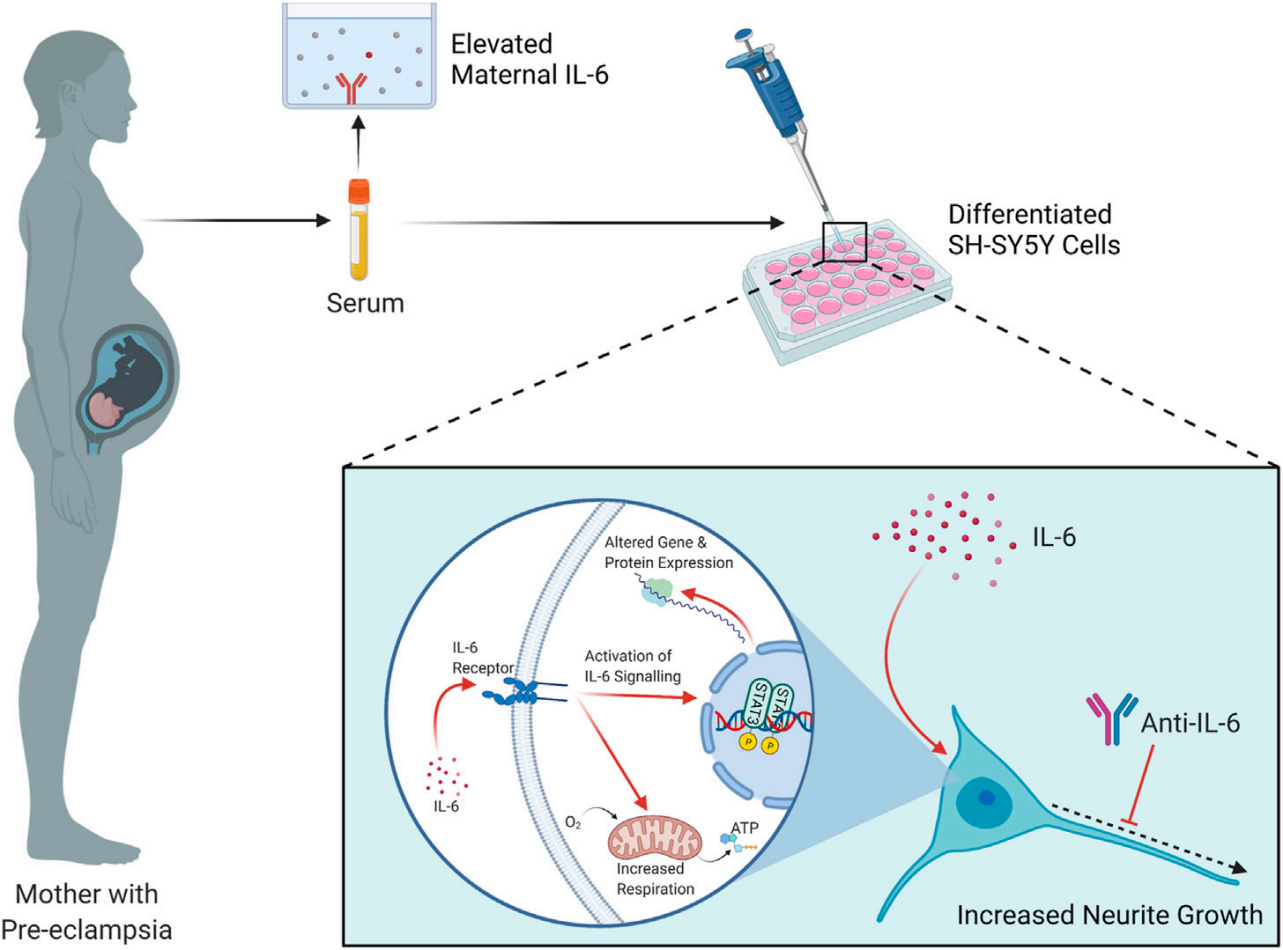
Summary of findings and proposed mechanism. Elevated IL-6 is detected in the circulation of pregnant women with Pre-eclampsia, compared to healthy pregnant controls. When applied to RA- or RA/BDNF-differentiated SH-SY5Y cells, this serum induces increases in neurite growth and mitochondrial oxygen consumption. It is proposed that the elevated IL-6 in maternal serum in PE activates the IL-6 signaling pathway in these cells, terminating in the phosphorylation and consequent induction of the transcription factor STAT3 which alters the gene expression profile of the cell, contributing in part to this respiratory and neurite growth increase phenotype. Schematic created with Biorender.com.

To investigate whether IL-6 alone is sufficient to induce the increased neurite growth and elevated OCR seen in PE serum-treated cells, RA-differentiated SH-SY5Y cells were treated with 20 ng/mL IL-6 daily for 72 h. IL-6 treatment increased neurite growth (t_3_ = 4.445, *p* < 0.05) (Figures 5A, D), did not affect oxidative stress (t_3_ = 0.3762, *p* = 0.732) (Figures 5B, E) and decreased cytotoxic cell membrane damage (t_3_ = 43.897, *p* < 0.05) (Figure 5C) after 72 h. To validate these findings in the more differentiated model, SH-SY5Y cells differentiated according to the 12-day RA/BDNF paradigm were treated with 20 ng/mL IL-6 daily for the last 3 days of differentiation. IL-6 treatment in this differentiation paradigm similarly increased neurite growth, although this was not statistically significant (t_3_ = 1.643, *p* = 0.1989) (Figure 5F), and did not affect oxidative stress (t_3_ = 0.4987, *p* = 0.652) (Figure 5G) or cytotoxicity (t_3_ = 1.422, *p* = 0.250) (Figure 5H). IL-6-induced neurite growth was completely prevented by anti-IL-6, with a significant main effect for IL-6 (F_1,4_ = 34.08, *p* < 0.01) (Figures 5I, J); *post-hoc* analyses showed IL-6 increased neurite growth in the absence of anti-IL-6 (*p* < 0.01), but not in its presence (*p* = 0.750). Collectively these data show that elevations in maternal IL-6 in PE (Figure 6). Likely mediates the neurite growth promoting effects of maternal PE serum on neurite growth in SH-SY5Y cells.

## 4 Discussion

Pre-eclampsia is a hypertensive disorder of pregnancy which is associated with an increased risk of neurodevelopmental disorders in affected offspring, although the mechanisms involved in this association are largely unknown. This study sought to characterize the effects of serum from women with pre-eclampsia on neuronal development at the single-cell level using neuronally-differentiated SH-SY5Y cells.

Before commencing human sera experiments, we initially validated the model of RA-induced neuronal differentiation by assessing the effects of RA on SH-SY5Y cells. RA treatment has previously been shown to increase protein expression of the neuronal markers MAP2, NeuN, and NSE (Lopes et al., 2010; Schneider et al., 2011), and here we observed significantly increased expression of the marker βIII tubulin. Similarly, the RA-induced elongation of neurites seen here is in line with previous reports (López-Carballo et al., 2002; Lopes et al., 2010; Teppola et al., 2016). RA-treated cells also exhibited a reduced capacity to migrate, which has been observed in a related SK-N-SH neuroblastoma cell line (Messi et al., 2008), and is demonstrative of a functional loss of neuroblastoma phenotype. Overall, these results provide evidence that cells exposed to RA are differentiating towards a neuronal phenotype. In all subsequent experiments, SH-SY5Y cells were differentiated either with RA for 72 h, or more prominently differentiated with RA and BDNF for 12 days.

Differentiated SH-SY5Y cells were then exposed to serum from women either with PE or a healthy uncomplicated pregnancy for 72 h. When compared to control serum-treated cells, those exposed to PE serum exhibited increased neurite growth and elevated mitochondrial function. This increased neurite growth is in line with observations from the one other study that performed a similar experiment in rat primary cortical neurons (Curran et al., 2018), demonstrating that the neurite growth induced by PE serum is conserved across *in vitro* models. The effect on OCR, however, is in contrast to that seen in human umbilical vein endothelial cells, where OCR was decreased following exposure to PE serum (McCarthy and Kenny, 2016). This illustrates how the effects of PE serum may be target cell-type specific, which is perhaps unsurprising considering that serum is a complex milieu of various ligands and that each cell type expresses a distinct pattern of receptors. However, these results suggest the presence of circulating maternal factors in PE which can directly affect neuronal development and the metabolism of neuronal-like cells differently to circulating factors of a healthy pregnancy.

PE is often accompanied by intra-uterine growth restriction (IUGR), which may obscure the relationship between PE and fetal brain development. However, a number of studies have demonstrated that, when restricting the study population to IUGR-exposed offspring, stratifying the data into average- or small-for-gestational-age offspring, or using mediation analyses, that PE still exerts an independent influence on the risk for neurodevelopmental disorders, due to some specific physiological feature (s) of PE (Many et al., 2003; Morsing and Maršál, 2014; Lahti-Pulkkinen et al., 2020; Basso et al., 2022). Importantly, none of the subjects in the control or PE group in the current study experienced IUGR, which could otherwise have confounded the interpretation of our results. Thus, any molecular factors observed to play a mechanistic role are likely to be due to pathophysiological changes intrinsic to PE, and not secondary to comorbid IUGR.

Considering IL-6/STAT3 signaling is known to have the capacity to modulate neurite growth and mitochondrial activity, we then investigated levels of IL-6 in patient sera and this was found to be elevated in PE, in agreement with previous reports in women with PE from other cohorts (Sharma et al., 2007; Aggarwal et al., 2019; Gencheva et al., 2021). Thus, it was of interest whether the IL-6 signaling pathway, which culminates in phospho-activation of STAT3, is stimulated in differentiated SH-SY5Y cells exposed to PE serum. Phosphorylation of STAT3 at Tyr_705_ was significantly higher in cells exposed to PE serum relative to control serum, which suggests increased activity of the IL-6 signaling pathway in these cells following exposure to PE serum. IL-6-STAT3 signalling activates several target genes that regulate cell survival and apoptosis, proliferation and differentiation, inflammation, as well as mitochondrial-associated genes (Carpenter and Lo, 2014), all of which can significantly affect neuronal development. Thus, developing neurons in the brain of offspring exposed to elevated maternal IL-6 in the context of PE may be driven towards an altered pattern of gene and protein expression, ultimately influencing their neurite growth, respiration, and developmental trajectory. Importantly, the increased neurite growth of differentiated SH-SY5Y cells was attenuated by IL-6 neutralization, demonstrating that IL-6 is *necessary* for this effect.

Differentiated SH-SY5Y cells were next treated with IL-6 for 72 h, and this induced a similar effect to PE serum. IL-6 treatment increased both neurite growth, a phenotype comparable to the difference between cells exposed to PE vs. control serum. These effects agree with previous studies from different neuronal models wherein neurite growth and mitochondrial activity were increased by IL-6 and/or STAT3 activity (März et al., 1997; Miao et al., 2006; Zhou and Too, 2011; Yang et al., 2012; Leibinger et al., 2013; Luo et al., 2016; Yang and Rincon, 2016; Su et al., 2020). Thus, IL-6 alone is also *sufficient* to augment neurite growth and mitochondrial respiration in differentiated SH-SY5Y cells. While we have shown here that PE serum, *via* IL-6, increases neurite growth and mitochondrial respiration, it is still unclear whether these are independent effects, or whether the increase in neurite growth is driving the elevated oxygen consumption due to an increased demand for ATP.

While the current study has shown that elevated IL-6 in PE increases neurite growth and mitochondrial respiration, it is likely to be one among several biomolecules altered in PE that can affect neurodevelopmental processes. The foetal brain is likely to be exposed to altered levels of various proteins, lipids, metabolites, microRNAs and other compounds in the context of PE, many of which could influence the developing brain, and this will be important to investigate in future work. Perhaps the most well-characterised molecular change in PE is an increase in placental-derived sFlt-1, which could impair feto-placental angiogenesis and the development of the fetal neurovascular unit (Torres-Vergara et al., 2022; Vogtmann et al., 2022), but whether sFlt-1 in PE directly affects neuronal development, as we shown here for IL-6, is less well known.

The approach described in this study of exposing cells to PE maternal serum as they develop neurites has allowed us to probe the cellular and molecular mechanisms of the consequences of PE exposure on developing neural cells. A significant strength of this work is the use of human sera, as circulating factors in animal or cell models of PE may differ from the serum profile of women with idiopathic pre-eclampsia. Significant strength is added to this study by the fact that the main result–increased neurite growth caused by PE serum–was replicated in differentiating human neural progenitor cells. Despite these advantages, there are however limitations and opportunities for future development of this work. Firstly, there are always inherent difficulties in extrapolating results from *in vitro* models to whole systems and processes like human neurodevelopment, albeit our aim was to study effects on single cells. Secondly, as we have shown that factors within maternal serum in PE can affect the parameters we investigated in this study, in future work it will be of equal interest to characterize the effects of PE placental secretions on neuron development. Additionally, there is one important question regarding the role of IL-6 signaling in this study–although the concentration of IL-6 is substantially higher in PE than control serum, it is still considerably lower than the concentration of recombinant IL-6 required to elicit the response in differentiated SH-SY5Y cells. There are a number of explanations for this, such as that there are other ligands elevated in the PE serum, such as IL-10 or IL-11, which also activate STAT3 signaling; that there are other circulating factors that sensitize the cells to the effects of IL-6; that other factors, acting through independent mechanisms have cumulative small effects that are only detectable when combined; or that IL-6 in the serum is acting partially through *trans*-signaling, an alternative and potent mechanism that involves binding of IL-6 to a soluble form of the IL-6 receptor (sIL-6Rα), which is absent when treating with IL-6 alone (Garbers et al., 2018). However, the key point remains: exposure to maternal PE serum elevates pSTAT3 signaling and changes neural cellular function, in an IL-6-dependent mechanism.

Overall, this study has shown that there are circulating factors in the serum of women with PE that increase neurite growth and mitochondrial respiration, two important neurodevelopmental parameters, in differentiated SH-SY5Y cells; that IL-6 is elevated in their sera and placenta, that this induces STAT3 phosphorylation in these cells; and that IL-6 alone is both necessary and sufficient for this phenomenon. We therefore propose that the elevated IL-6 is responsible, at least partially, for these effects (Figure 6). This may have important implications for our understanding of the physiological relationship between pre-eclampsia and neurodevelopment *in vivo*, considering IL-6 is able to permeate both the human placenta and the blood-brain barrier (Zaretsky et al., 2004; Banks, 2005), and IL-6 is correspondingly elevated in the circulation of human neonates born to pre-eclamptic pregnancies (Tosun et al., 2010) and the brains of rat pups exposed to a pre-clinical model of PE (Giambrone et al., 2019). This suggests IL-6 as a potential pathway for early therapeutic intervention, not to prevent the progression of PE in the mother, but to attenuate its deleterious effects on the fetal brain, although further preclinical and clinical studies will be required to discern this. These data provide important insights into our understanding of the consequences of pre-eclampsia exposure and its effects on neurodevelopmental processes which may influence neurodevelopmental trajectories in exposed offspring.

## Data Availability

The raw data supporting the conclusions of this article will be made available by the authors, without undue reservation.

## Supplementary material

Supplementary Figure S1 RA Promotes Neuronal Differentiation in SH-SY5Y Cells. SH-SY5Y cells were treated with 10 µM RA daily for 72 h. βIII Tubulin protein expression measured by immunoblotting relative to GAPDH expression. **(C)** Photomicrographs of SH-SY5Y cells stained for βIII Tubulin by immunocytochemistry, with or without RA. **(D)** Representative photomicrographs and **(E)** graph of neurite growth following 72 h treatment with RA. Cells are stained with the vital cell dye Calcein-AM. **(F)** Graph and **(G)** representative photomicrographs of cell migration measured by wound width at 24 h, 48 h, and 72 h post-scratch relative to initial wound width. Data are mean + SEM from four independent experiments (*n* = 4) for B and F, or eight independent experiments (*n* = 8) for E, all expressed as percentage of the control*.* [**p* < 0.05; ***p* < 0.01; *** *p* < 0.001; **** *p* < 0.0001 vs. control. One-way ANOVA and *post-hoc* Dunnett’s multiple comparisons test for B; Student’s unpaired *t*-test for E; two-way ANOVA and *post-hoc* Fisher’s least significant difference (LSD) for F].

Supplementary Figure S2 Pre-eclampsia serum does not affect mitochondrial superoxide, biomass or membrane potential in differentiated SH-SY5Y Cells. RA-differentiated SH-SY5Y cells were treated with 3% (v/v) serum from pre-eclamptic patients (PE) or normotensive pregnant controls for 72 h Mean fluorescence intensity of **(A)** MitoSOX^TM^ Red, **(B)** MitoGreen, or **(C)** MitoTracker^TM^ Red FM, as measures of mitochondrial superoxide, biomass and membrane potential, respectively. Data are mean + SEM from four serum samples per group for A-C (*n =* 4), expressed as percentage of the control. (Student’s unpaired *t*-test).

Supplementary Figure S3 Comparison of ReNcell® VM neural progenitor cells at day 1 vs. day 7 *in vitro*. ReNcell® VM neural progenitor cells were differentiated for 7 days by removal of EGF and FGF2 from the culture medium. Blue = DAPI, green = βIII tubulin, red = GFAP, PhC = phase contrast microscopy.

## Abbreviations

ADHD, attention-deficit/hyperactivity disorder; ASD, autism spectrum disorder; ATP, adenosine triphosphate; BDNF, brain-derived neurotrophic factor; BMI, body mass index; BP, blood pressure; BSA, bovine serum albumin; DMEM, dulbecco’s modified eagle’s mixture; E, embryonic day; EGF, epidermal growth factor; FBS, fetal bovine serum; FCCP, carbonyl cyanide-p-trifluoromethoxyphenylhydrazone; FGF2, fibroblast growth factor 2; ID, intellectual disability; IL-6, interleukin-6; LDH, lactate dehydrogenase; MIA, maternal immune activation; OCR, oxygen consumption rate; PBS, phosphate-buffered saline; PE, pre-eclampsia; PFA, paraformaldehyde; RA, retinoic acid; SDS-PAGE, sodium dodecyl sulphate polyacrylamide gel electrophoresis; STAT3, signal transducer and activator of transcription 3; TBS, Tris-buffered saline.
