# Supplementary figures and images for: Maternal pre-eclampsia serum increases neurite growth and mitochondrial function through a potential IL-6-dependent mechanism in differentiated SH-SY5Y cells
Source: Front Physiol. 2023 Jan 12;13:1043481. doi: 10.3389/fphys.2022.1043481 (PMC9877349; doi:10.3389/fphys.2022.1043481)

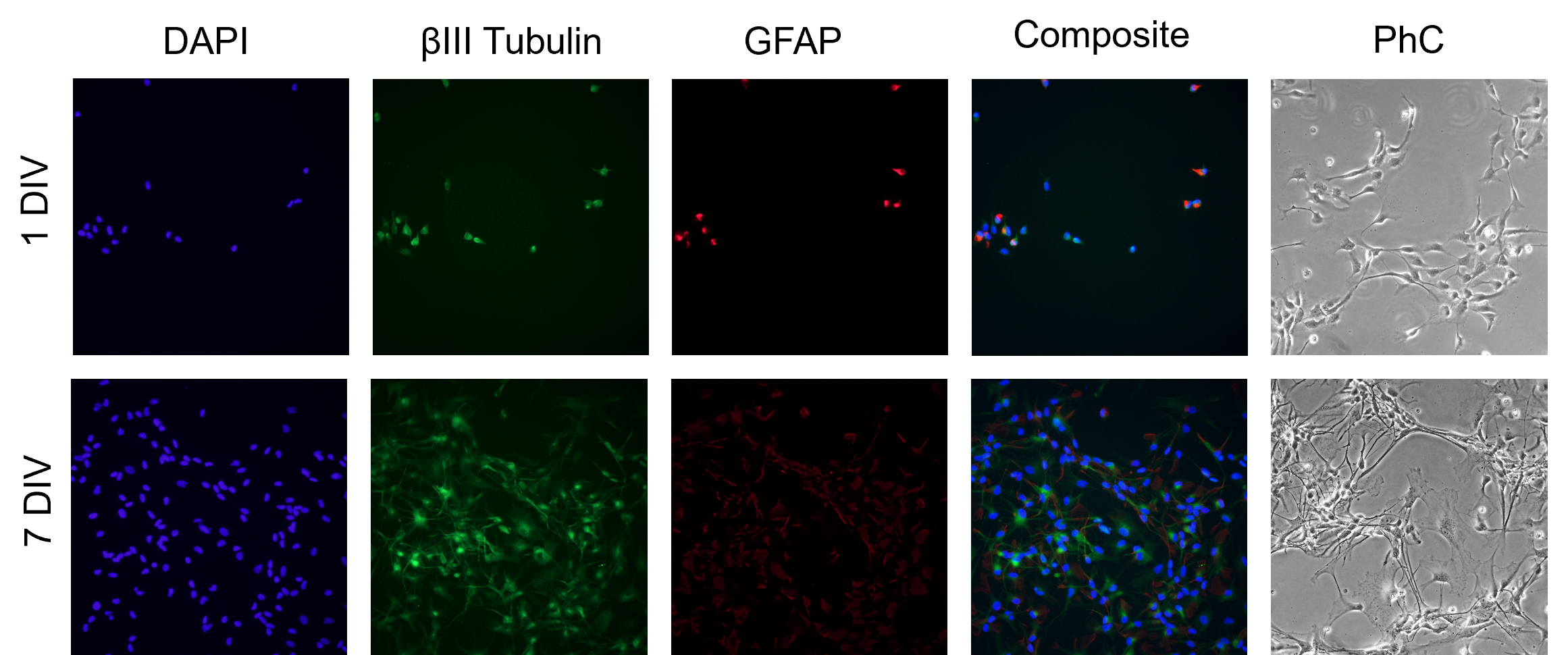

Supplement: Supplementary file 1 [file Image3.tif]

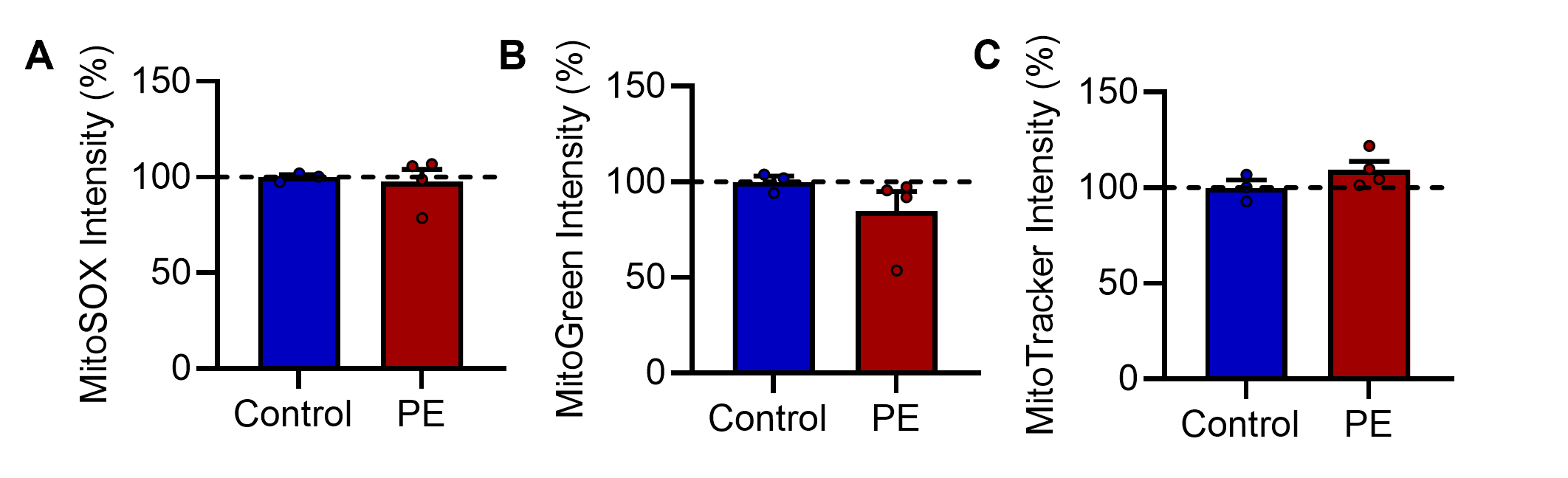

Supplement: Supplementary file 2 [file Image2.tif]

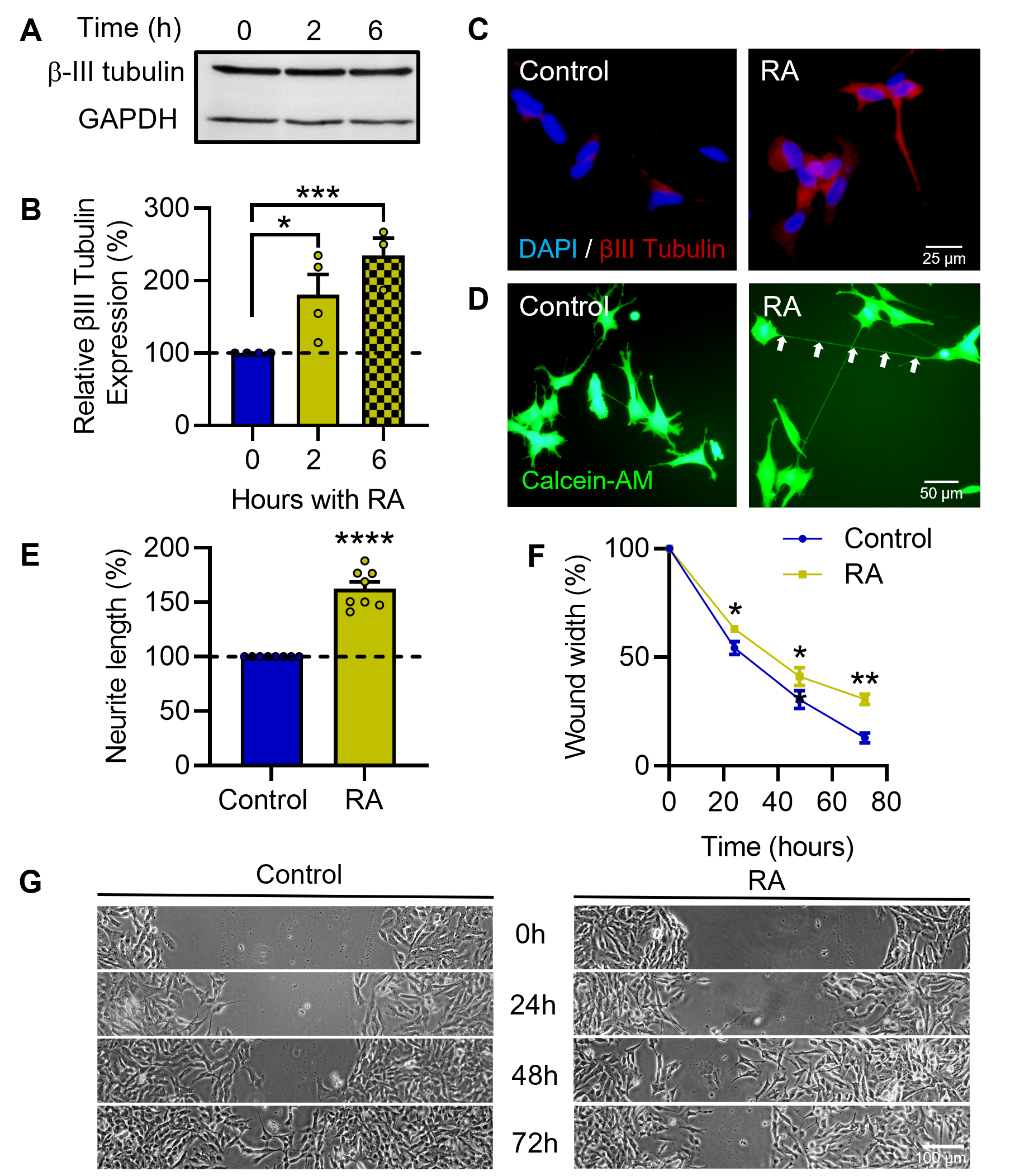

Supplement: Supplementary file 3 [file Image1.tif]
